# A new perspective on calmodulin-regulated calcium and ROS homeostasis upon carbon black nanoparticle exposure

**DOI:** 10.1007/s00204-021-03032-0

**Published:** 2021-03-27

**Authors:** Nisha Verma, Mario Pink, Simone Schmitz-Spanke

**Affiliations:** Institute and Outpatient Clinic of Occupational, Social and Environmental Medicine, University of Erlangen-Nuremberg, Henkestrasse 9-11, 91054 Erlangen, Germany

**Keywords:** Carbon black nanoparticles, Reactive oxygen species (ROS), Calcium homeostasis, Mitochondrial ROS

## Abstract

Toxicological studies propose that exposure to carbon black nanoparticles induces organ injuries and inflammatory responses. Besides, current understanding of the molecular mechanisms implies that carbon black nanoparticles (CBNP) exposure induces the production of reactive oxygen species (ROS) causing inflammation, mitochondrial dysfunction or disturbance in calcium homeostasis. However, the precise mechanisms whereby CBNP exert these effects in the lung are still not fully understood. To gain insight into the possible mechanism of CBNP exerted toxicity, human alveolar epithelial cells (A549) were exposed to different concentrations of CBNP and for different timepoints. The reaction of the cells was monitored by the systematic use of cell-based measurements of calcium and ROS, in the presence and absence of calcium (Ca^2+^) pump inhibitors/chelators and antioxidants. Followed by an in-depth PCR analysis of 84 oxidative stress-related genes. The measurements revealed, as compared to the control, that exposure to CBNP nanoparticles leads to the generation of high ROS levels, as well as a disturbance in calcium homeostasis, which remained primarily unchanged even after 24 h of exposure. Nevertheless, in presence of antioxidants *N*-acetylcysteine (NAC) and Trolox, ROS formation was considerably reduced without affecting the intracellular calcium concentration. On the other hand, Ca^2+^ pump inhibitors/chelators, BAPTA (1,2-bis(o-amino phenoxy)ethane-N, N, N′, N′-tetraacetic acid) and verapamil not only decreased the Ca^2+^ overload, but also further decreased the ROS formation, indicating its role in CBNP-induced oxidative stress. Further, a PCR array analysis of A549 cells in presence and absence of the calmodulin (CaM) antagonist W7, indicated toward nine altered oxidative stress-related genes which further confirmed our cytotoxicity results. Obtained data suggested that CBNP exposure elevates calcium ion concentration, which further contributes to oxidative stress, via the calcium-binding protein CaM. Its inhibition with W7 leads to downregulation in gene expression of nine oxidative stress-related genes, which otherwise, as compared to control, show increased gene expression. The results of the study thus confirm that exposure of lung epithelial cells to CBNP leads to oxidative stress; however, the oxidative stress itself is a result of a disturbance in both calcium and ROS homeostasis, and should be considered while searching for a new strategy for prevention of CBNP-induced lung toxicity.

**Supplementary Information:**

The online version contains supplementary material available at 10.1007/s00204-021-03032-0.

## Introduction

Scientific and industrial attainments within the last few years have led to discoveries in nanotechnology which were far beyond the imagination of mankind half a century ago (Jeevanandam et al. 2018). Globally, scientists are still discovering the unique properties of daily used materials at the sub-micrometer range domain. Among others, carbon black nanoparticles (CBNP) are identified as one of the most industrial manufactured chemicals due to their widespread applications in automobile, printing, and paint industry (Uddin 2017). Studies suggest that on average, workers encounter approximately one million tons of CBNP (used as raw material) thus raising serious health concerns (Sharma 2010). Due to their particulate size, these particles can be easily dispersed in the ambient conditions and can be readily inhaled thus causing lung diseases (Vesterdal et al. 2010) (Saputra et al. 2014; Umezawa et al. 2018).

Particularly, exposure to high concentrations of nanoparticles is known to impair lung clearance by macrophages (Gustafson et al. 2015). The overloading of the lung thus can initiate a severe inflammatory response, which ultimately leads to downstream events like lung fibrosis, and upstream events such as oxidative stress (Gustafson et al. 2015).

Oxidative stress is one of the most known and reported toxicities which the cells encounter when exposed to nanoparticles (Niranjan and Thakur 2017). High ROS levels thus generated are known further to cause severe cell damage which ultimately leads to cell death. However, often this switch is mediated by calcium signaling (Huang et al. 2017). Disturbance in calcium homeostasis upon nanoparticle exposure has been reported in many studies, (Holme et al. 2019; Prada et al. 2017), but a mutual interplay of ROS and calcium ions upon nanoparticle exposure has hardly been explored (Stone et al. 2000). Increasing evidence suggests that interactions between calcium and ROS are necessary for signaling and proper functioning of cellular signaling networks (Görlach et al. 2015; Hempel and Trebak 2017; Yan et al. 2006).

The current study, therefore, explores this interplay of ROS and calcium signaling upon CBNP exposure to understand the pathomechanism behind the CBNP-induced lung toxicity. To achieve our aim, a systematic analysis of disturbance in calcium and ROS homeostasis upon CBNP exposure was carried out in human alveolar epithelial cell line (A549). The cells were exposed to well-characterized and commercially available carbon black nanoparticle Printex 90 in presence and absence of Ca^2+^ pump inhibitors/chelators and antioxidants. Concentration ranging from 2 to 250 µg/mL and exposure time of 3, 6 and 24 h were tested to comprehensively evaluate the response of CBNP in A549 cells. Finally, to understand the interplay of ROS and calcium signaling at the molecular level, a PCR array analysis of genes involved in oxidative stress, in presence and absence of calcium-regulated protein calmodulin (CaM) was carried out.

## Material and methods

### Reagents

Standard chemicals for cell culture (fetal calf serum (FCS), penicillin/streptomycin and L-glutamine) were purchased from c.c.pro (Oberdorla, Germany). Carbon black (Printex 90) was provided by Evonik (former Degussa, Germany). The fluorescent dyes 2′,7′-dichlorodihydrofluorescein diacetate (H_2_DCFDA), Fluo-4/AM and Rhod-2/AM and MitoSox Red were acquired from Invitrogen™ (Darmstadt, Germany). RNeasy Plus Mini Kit, RT^2^ First Strand Kit and the Human Oxidative Stress Plus RT2 Profiler PCR array (PAHS-065Z) were purchased from (Qiagen, Hilden, Germany).

## Cell culture and carbon black nanoparticle exposure

The human alveolar epithelial cells (A549, ATCC® CCL-185™) were cultured until confluency in Dulbecco’s Modified Eagle Medium, supplemented with 10% (v/v) FCS, 7.4 mg/mL L-glutamine, 100 units/mL penicillin, and 100 mg/mL streptomycin at 37 °C in a humidified atmosphere of 95% air and 5% CO_2_. After 24 h of culture, cells were exposed to varying concentration of CBNP for further 3, 6 and 24 h.

## Preparation of particles

CBNP were weighed and suspended in the culture media to obtain a stock solution of 1 mg/mL. The stock solution was then sonicated in a water bath for 5 min. The nanoparticles suspension obtained was subsequently diluted 1:2 (v/v) with the cell culture medium to achieve the nominal concentrations. The nanoparticles dilutions were sonicated to distribute the nanoparticles as homogeneously as possible, before adding them to the cells.

## Determination of hydrodynamic size and zeta potential of CBNP

The particle size distribution of CBNP was measured using dynamic light-scattering (Nano Zetasizer ZS; Malvern Instruments, Worcestershire, UK) after suspending the nanoparticles in the cell culture medium, using an ultrasonic water bath for 5 min at room temperature. Zeta potential values were obtained using the Smoluchowski model for analysis. In brief, 1 mL dispersions of CBNP (100 μg/mL) in culture media were prepared in cuvettes and analyzed after 24 h of dispersion. The samples were analyzed in triplicates. Calibration and blank samples were analyzed prior to all measurements.

## Determination of cellular reactive oxygen species (ROS) level

The production of ROS by A549 cells was measured using the fluorescent dye 2′,7′-dichlorodihydrofluorescein diacetate (H_2_DCFDA; Invitrogen). For the experiment, 25,000 cells/mL were seeded into 96-well plates. 24 h after seeding, cells were exposed to different CBNP nominal concentrations ranging from 2 to 250 µg/mL for 3, 6 and 24 h. Cells treated with cell culture medium were used as negative control, while 100 μM of H_2_O_2_ served as a positive control. For ROS measurement, the cells were then washed with HBSS buffer (pH 7.2), and incubated with 10 μM H_2_DCFDA in HBSS (pH 7.2) for 30 min at 37 °C. The fluorescence intensity was measured at an excitation wavelength of 485 nm and an emission wavelength of 535 nm.

In a separate series of experiments, to determine whether ROS generation due to CBNP exposure in these cells was responsible for increased calcium concentrations, calcium measurements (as described below) were performed in the cells pre-treated to antioxidants- 6‐hydroxy‐2,5,7,8‐tetramethylkroman‐2‐carboxyl acid (Trolox) and *N*-acetylcysteine (NAC). A water-soluble vitamin E analog, Trolox confers protection due to its intracellular scavenging activity (Hamad et al. 2010), whereas, NAC is a synthetic acetylated derivative of amino acid cysteine, and because of its sulfhydryl group acts as a free radical scavenger (Aldini et al. 2018). For the experiment, the cells were preincubated with Trolox (35 μM) or NAC (50 μM) for 1 h, the incubation medium was then removed, to further incubate cells with CBNP at a final concentration of 125 mg/mL for another 24 h. 125 µg/mL concentration of CBNP was chosen because it showed maximum damage, as compared to the control cells. Calcium measurements were then carried out according to the protocols described in the paper.

## Determination of intracellular calcium concentration

To study CBNP-induced changes in calcium homeostasis in different cellular compartments, two Ca^2+^-sensitive dyes were used: Fluo-4/AM to observe changes in intracellular calcium concentration ([Ca^2+^]_*i*_), and Rhod-2/AM to determine alterations in mitochondrial calcium concentration. 25,000 cells/mL were seeded onto 96 well plates. After 24 h culture, cells were exposed to nominal concentrations of CBNP as described above for 3, 6 and 24 h. After the exposure, cells were washed twice with HBSS (pH 7.2) and incubated with acetoxymethyl (AM) ester of Fluo-4/AM (1.4 µM) and Rhod-2/AM (3.6 µM) in the same buffer for 30 min at room temperature (RT). The incubation was carried out RT to reduce the subcellular compartmentalization of the dyes, an inherent problem with the AM ester loading techniques. The cells were then further incubation for another 30 min at 37 °C to allow complete de-esterification of intracellular AM esters. Subsequently, the dye solution was removed, and the cells were incubated for 30 min at 37 °C in HBSS (pH 7.2) (3 mL) supplemented with 2.5 mM probenecid to decrease dye leakage. For calcium measurements, the cells were washed once with pre-heated HBSS (37 °C), and the measurement was performed in same buffer containing probenecid. The green fluorescence of Fluo-4 was excited at 488 nm and was collected through a 505–535 nm bandpass filter, whereas the red fluorescence of Rhod-2, excited at 543 nm, was collected through a 560 nm long-pass filter.

To determine whether the increase in calcium ions upon CBNP exposure had any effects on ROS levels and its signaling, A549 cells were pretreated with the calcium channel blocker verapamil (50 μM), calcium chelator BAPTA-AM (1,2-bis(*o*-amino phenoxy)ethane-*N*, *N*, *N*′, *N*′-tetraacetic acid)) (25 μM) and calmodulin antagonist W-7 (*N*-(6-Aminohexyl)-5-chloro-1-naphthalene sulfonamide) (5 µM) for 1 h. The incubation medium was then removed, and the cells were further incubated with CBNP at a nominal concentration of 125 mg/mL for another 24 h. ROS measurements were then carried out according to the protocols described.

## Detection of the mitochondrial membrane potential (MMP)

For the determination of the mitochondrial potential, A549 cells were seeded at a concentration of 25,000 cells/mL in 96-well plates for 24 h. After 24 h culture, the cells were exposed to nominal concentrations of CBNP as described above for 3, 6 and 24 h. After the exposure, cells were washed twice with HBSS buffer (pH 7.2) and incubated with a cationic dye JC-1 (5,5′,6,6′-tetrachloro-1,1′,3,3′-tetraethyl benzimidazolocarbo-cyanine iodide, 10 µM) in HBSS (pH 7.2) for 30 min at 37 °C. Cells treated with normal cell culture medium were used as negative controls, while 100 μM of the ionophore valinomycin served as positive control. Following incubation with the dye, the cells were washed three times with pre-heated (37 °C) HBSS (pH 7.2) and measured immediately. Measurements were obtained as a ratio of red aggregate of JC-1 dye with absorption/emission at 585/590 nm /green aggregate of the dye, with absorption/emission of 510/527 nm in the mitochondria, using the Tecan microplate reader (Tecan, Mainz, Germany).

## Determination of mitochondrial ROS using MitoSOX Red

Alterations of the mitochondrial ROS levels due to CBNP were measured using a mitochondria-targeted superoxide indicator (MitoSOX Red) dye. Briefly, 25,000 cells/mL of A549 cells were seeded in 96-well plates for 24 h. After 24 h culture, the cells were exposed to nominal concentrations of CBNP, as described above for 3, 6 and 24 h. After the exposure, the cells were washed twice with pre-heated HBSS buffer (37 °C, pH 7.2) and further incubated with the same buffer containing MitoSOX Red (5 µM, 10 min, 37 °C). Following incubation with the dye, the cells were washed three times with pre-heated HBSS (37 °C, pH 7.2) and measured immediately using the Tecan microplate reader (Tecan, Mainz, Germany) at an excitation wavelength of 510 nm and an emission wavelength of 580 nm.

## RNA isolation

Total RNA from exposed and unexposed A549 cells (1.5 × 10^6^/mL) was isolated using the Qiagen’s RNeasy Plus Mini Kit (Qiagen, Hilden, Germany). Experiments were performed considering four replicates. Genomic DNA was removed from each sample by treatment with rDNase at 37 °C for 20 min (Qiagen, Hilden, Germany). RNA quality was assessed using analysis on a 2% agarose gel, while the concentrations were determined using a NanoDrop 2000 spectrophotometer (Thermo Fisher Scientific). The 260 nm/280 nm absorbance ratios of all samples were determined to be > 1.8. An aliquot of RNA (1 µg) was copied into cDNA using an RT^2^ First Strand Kit (Qiagen, Hilden, Germany). The pathway-focused Human Oxidative Stress Plus RT^2^ Profiler PCR array (Qiagen, 96-well format, catalog no. 35802175370698587056PAHS-065Z) with 84 oxidative stress-associated genes (Supplementary Table 1) was used to assess the exposure-induced differential gene expression with an ABI 7500 real-time qPCR system (Thermo Fisher Scientific). In brief, 1 µg of cDNA was mixed with SYBR Green master mix provided with the kit and dispensed into each well of the PCR array plate containing the pre-dispensed gene-specific primer sets. The PCR was performed according to the manufacturer’s instructions. Each PCR array plate contained five housekeeping genes (Actb, Gapdh, Hsp90ab1, Hprt1, Gusb) for normalization of the PCR array data, and one negative control to monitor for genomic DNA contamination. The PCR array also contained three wells of reverse transcription controls (RTC) to verify the efficiency of the RT reaction and replicate positive PCR controls (PPC) to check the efficiency of the polymerase chain reaction, as well as a test for inter-well and intra-plate consistency.

## Data analysis

A comparative or ΔΔCt method of qPCR data analysis was performed on RT^2^Profiler PCR Array Data Analysis Webportal (https://www.qiagen.com). Transcriptional changes in cells exposed to CBNP were compared to changes in cells pretreated with CaM to assess its effects on CBNP-induced oxidative stress. Differentially expressed genes were identified using Qiagen RT^2^ Profiler Data Analysis software, with significance defined as *P* < 0.05. The *P*-value adjusted for false discovery rate (FDR) was estimated to be 0.0005 (α/n; *α* = 0.05, and *n* = 84 genes). Bioinformatics tool, Search Tool for Interacting Chemicals (STRING) (http://string-db.org/) (von Mering et al. 2003) was used to elucidate the biological pathways associated with the individually identified genes.

## Statistical analysis

All tests were performed in at least four independent experiments. The level of statistical significance relative to control was calculated using the *t*-test. A *P* value of < 0.05 was significant.

## Results

### Determination of CBNP size and Z-potential

The hydrodynamic number-based size distribution revealed a narrow, unimodal peak with an average diameter below 150 nm for all particle suspensions. The zeta potential was recorded as − 8.92 mV to − 10.7 mV.

## Effect of CBNP exposure on cellular ROS levels

**Fig. 1:**
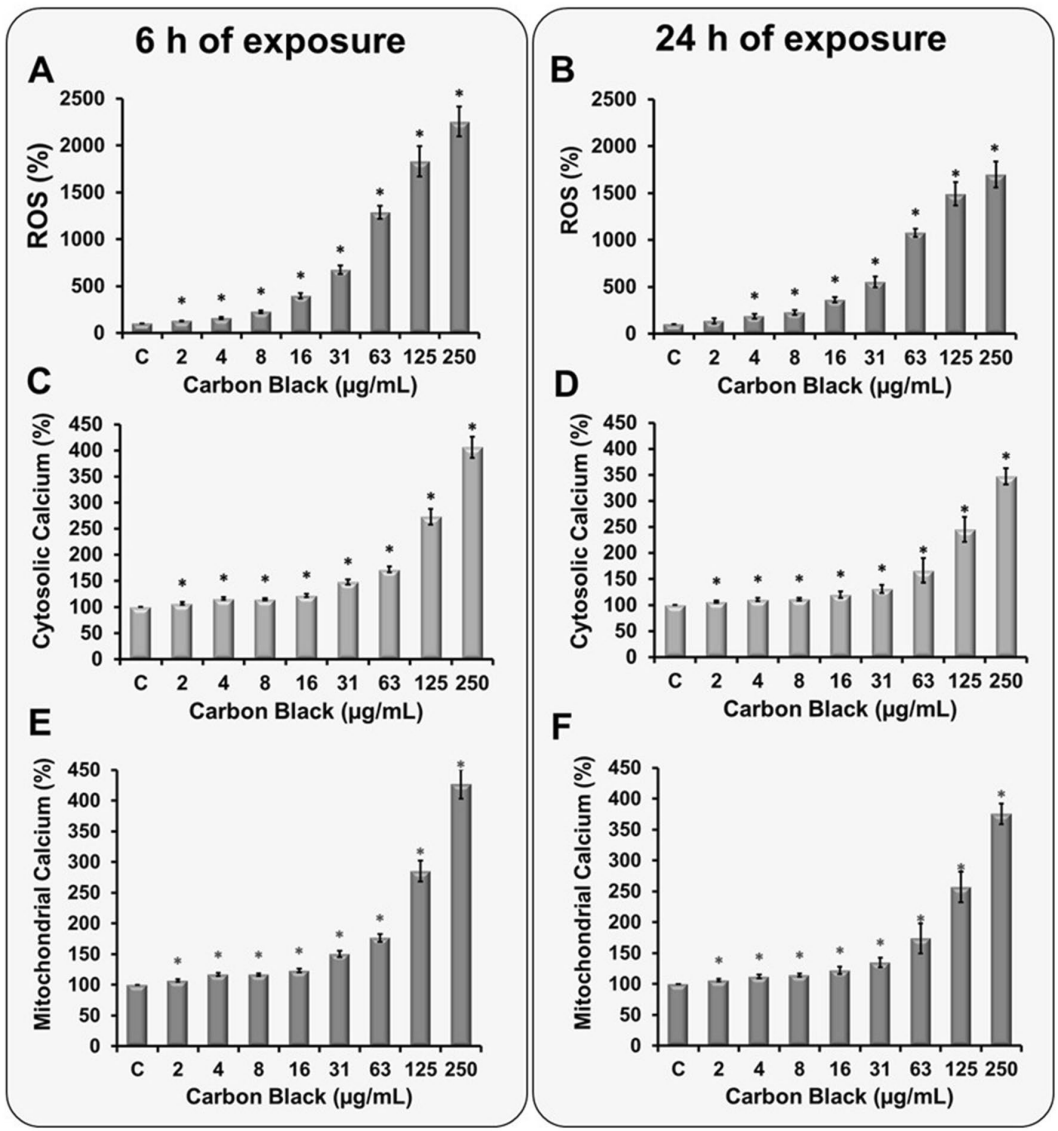
Alterations in cellular ROS and calcium homeostasis after exposure to CBNP. ROS measurements were made in cells exposed to different concentrations of CBNP (2 to 250 µg/mL) for 6 (**a**) and 24 h (**b**) using fluorescent dye H_2_DCFDA. H_2_O_2_ (100 µM) was used as a positive control. Data are expressed as the percentage of ROS levels in exposed cells as compared to unexposed cells arbitrarily set to 100%. Spectrofluorometric cytosolic (**c**—6 h, **d**—24 h) and mitochondrial (**e**—6 h, **f**—24 h) calcium measurements were made in A549 cells exposed to 2 to 250 µg/mL CBNP using Fluo-4/AM and Rhod-2/AM dyes. Data are expressed as the percentage of calcium levels found in treated cells as compared to untreated cells arbitrarily set to 100%. The data are presented as mean ± standard deviation of four independent experiments. The level of significance relative to the control was determined using *t*-test (*P < 0.05, ***P < 0.001)

Induction of reactive oxygen species upon nanoparticle exposure is considered one of the primary cause of nanoparticles induced toxicity (Fu et al. 2014). A549 cells after 3 h exposure (Supplementary Fig. 1A), showed minor cellular ROS alterations. However, after 6 h of exposure (Fig. 1a) at a concentration of 16 µg/mL onwards, a strong concentration-dependent increase in ROS formation with levels ~ 20–25 times higher than control (in cells exposed to 250 µg/mL CBNP) was observed. Even after 24 h of CBNP exposure (Fig. 1b), high ROS levels were maintained although slightly reduced as compared to 6 h exposure (~ 8–18 times higher than control).

## Changes in calcium homeostasis upon CBNP exposure

Deregulation in Ca^2+^ homeostasis upon nanoparticle exposure has been reported in many studies. After 3 h exposure, reasonable increase (~ 15%) in cytosolic and mitochondrial Ca^2+^ was observed already at moderate exposure concentrations (Supplementary Fig. 1B). Like ROS measurements, after 6 h of CBNP exposure at a concentration of 16 µg/mL onwards, a concentration-based increase in cytosolic and mitochondrial Ca^2+^ (Fig. 1c, e) was observed. In contrast to ROS, after 24 h exposure, the cellular calcium levels remained primarily unchanged compared to the 6 h results, 3.5 times as compared to the control (Fig. 1d–f).

## The interplay of cellular ROS and Ca^2+^upon CBNP exposure

**Fig. 2:**
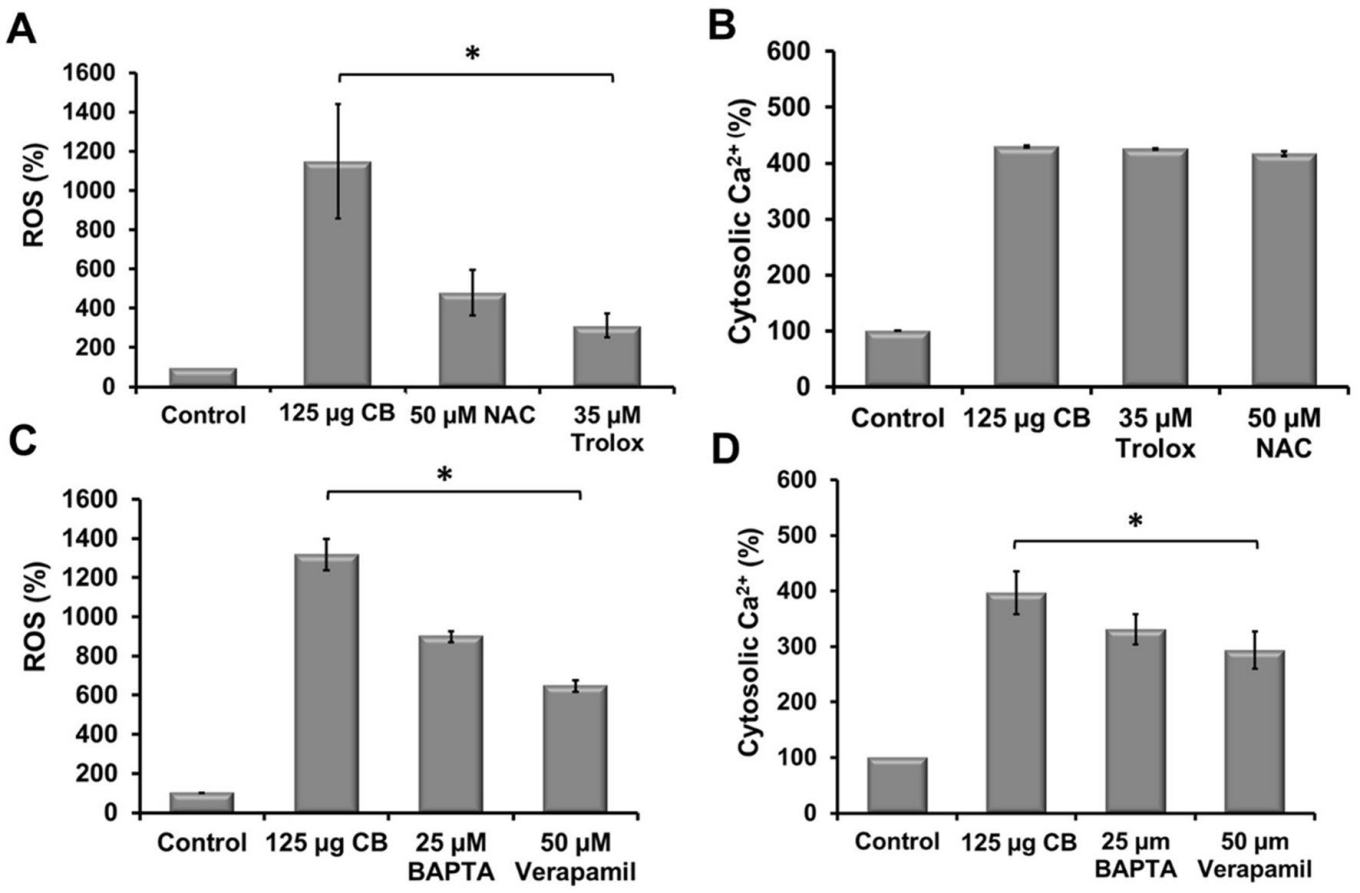
Interaction between ROS and Ca^2+^ upon CBNP exposure. CB exposed cells showed increased levels of ROS and intracellular Ca^2+^. **a** The antioxidants N-acetylcysteine (NAC) and Trolox reduced ROS levels, without affecting the intracellular calcium concentration **b**. On the other hand, **c** Ca^2+^ pump inhibitors/chelators not only decreased the Ca^2+^ overload, but also further decreased ROS levels **d**, indicating its role in CB-induced oxidative stress. The data is presented as mean ± standard deviation of four independent experiments. The level of significance relative to the carbon black exposure was determined using *t*-test (*P < 0.05, ***P < 0.001)

An interplay between ROS and calcium ions has been reported upon exposure to nanoparticles (Stone et al. 2000). In A549 cells, a modulation of intracellular ROS and calcium levels upon CBNP exposure was also observed. To analyze this interdependence in A549 cells, ROS measurements were carried out in presence of Ca^2+^ pump inhibitor verapamil, calcium chelator BAPTA and calcium measurements in presence of antioxidants NAC and Trolox (Supplementary Fig. 2). A significant increase in ROS levels was detected in cells treated with CBNP. When the cells were pretreated with NAC and Trolox, the production of ROS induced by CBNP was effectively reduced to 58% and 73%, respectively. Thus, suggesting that cytotoxic effect of CBNP in A549 cells to a great extent was exerted by ROS generation (Fig. 2a); however, it had no effect on intracellular calcium concentration (Fig. 2b). In the second set of experiments, Ca^2+^ pump inhibitors/chelators, BAPTA and verapamil pretreatment of the cells not only decreased the Ca^2+^ overload by 17% and 26%, respectively, (Fig. 2d) but also further decreased ROS levels by 35% in BAPTA treated cells, and 51% in verapamil pretreated cells (Fig. 2c). The results of this experiment indicated that an increase in calcium levels upon CBNP exposure involved the mobilization of calcium from both intracellular stores and extracellular influx, but most importantly contributed to the increased ROS levels upon CBNP exposure.

## The possible role of calmodulin

**Fig. 3:**
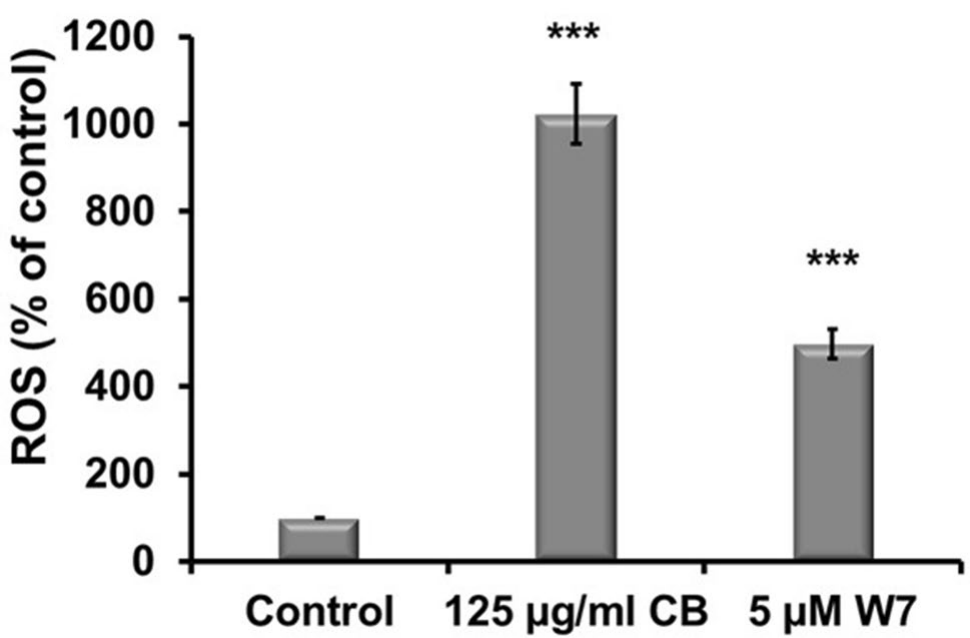
Effect of calmodulin inhibition on ROS levels. To elucidate the role of CaM in the interplay between ROS and calcium, ROS measurements were carried out in the presence and absence of CaM inhibitor W-7. A549 cells were cultured on a clear bottom 96 well plate and were pre-exposed to calcium antagonist, W-7 (*N*-(6-Aminohexyl)-5-chloro-1-naphthalene sulfonamide) (5 µM) for 1 h. The incubation medium was then removed, and cells were further incubated with CBNP at a nominal concentration of 125 mg/mL for another 24 h. ROS measurements were then made at an excitation wavelength of 485 nm and an emission wavelength of 535 nm. The data are presented as mean ± standard deviation of four independent experiments. The level of significance relative to the carbon black exposure was determined using *t*-test (*P < 0.05, ***P < 0.001)

To elucidate the role of CaM in the interplay between ROS and calcium, ROS measurements were carried out in the presence and absence of CaM inhibitor W-7. The exposure of A549 cells to CBNP (125 µg/mL) led to 15 times more ROS in exposed cells as compared to control, however upon blocking of CaM by its inhibitor W-7, the exposed cells exhibited a sharp drop in ROS levels by 50%. Thus, indicating its role, and thereby of calcium, in the regulation of ROS generated upon CBNP exposure in a CaM dependent manner (Fig. 3).

## Analysis of mitochondrial function upon CBNP exposure

**Fig. 4:**
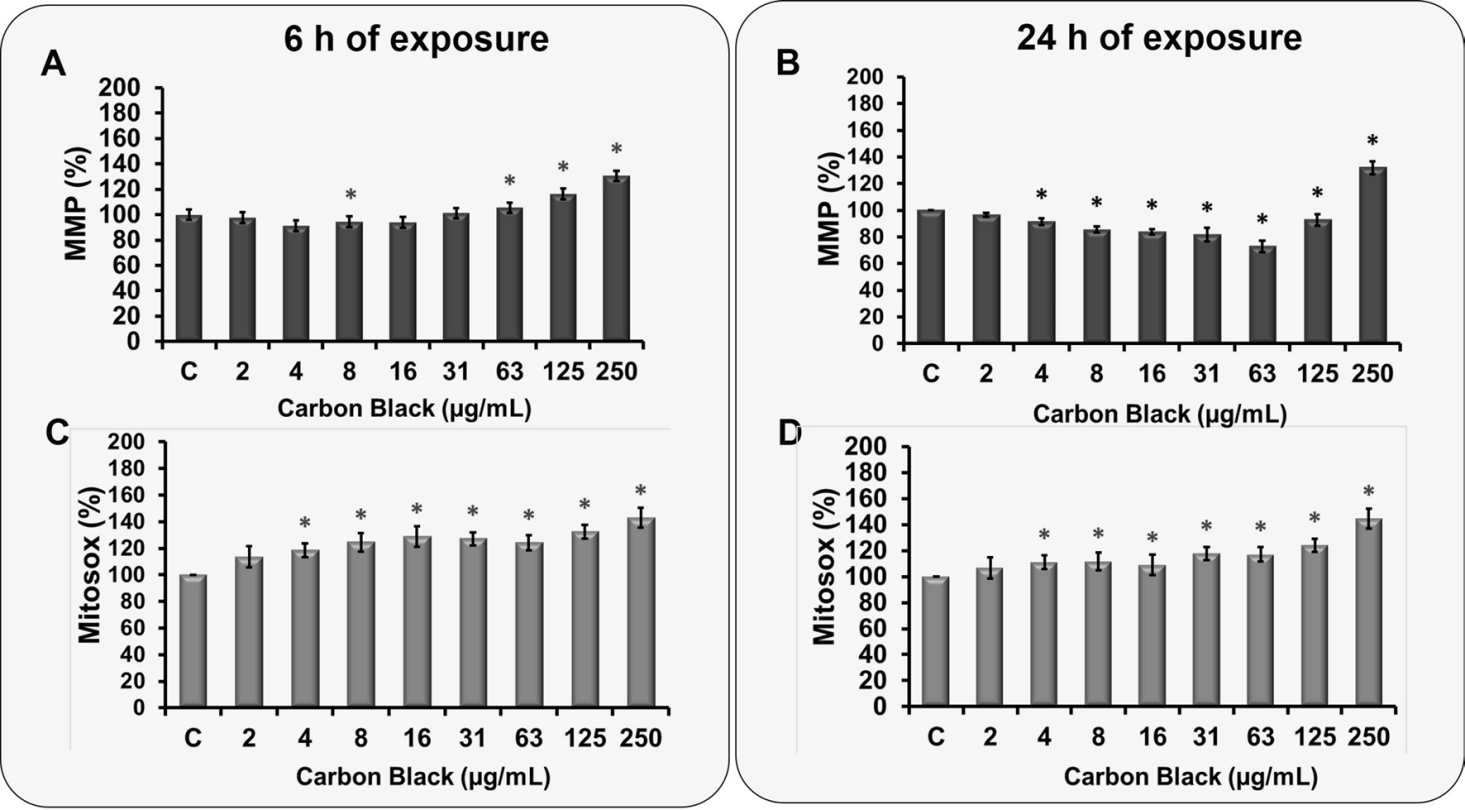
Changes in mitochondrial membrane potential and generation of mitochondrial ROS on CBNP exposure. **a** Determination of MMP on A549 cells using the JC-1 dye. Cells were cultured on a clear bottom 96-well plates with a clear bottom and were exposed to 2–250 µg/mL of CBNP for different time periods (6 and 24 h). Valinomycin (100 µM) was used as a positive control, while at the negative control, cells were exposed to culture medium. JC-1 dye (5 µM) was applied for 30 min. Measurements were obtained immediately as a ratio of a red aggregate of JC -1 dye with absorption/emission at 585/590 nm /green aggregate of the dye, with absorption/emission of 510/527 nm in the mitochondria using the Tecan microplate reader (Tecan, Mainz, Germany). **b** Fluorogenic dye MitoSox Red was used to determine the mitochondrial ROS generated upon CBNP exposure, A549 cells were incubated with a buffer containing 5 µM MitoSOX Red for 10 min. The measurements were then carried out at an excitation wavelength of 510 nm and an emission wavelength of 580 nm. The data is presented as mean ± standard deviation of four independent experiments. The level of significance relative to the positive control was determined using *t*-test (***P < 0.001)

As visible in Supplementary Fig. 1C, during the initial 3 h of exposure, the MMP of the cells remained unaltered. However, after 6 h exposure, as indicated by the increase of red to green ratio, the cells exhibited hyperpolarization of the mitochondria at higher concentration of CBNP exposure (63 to 250 µg/mL exposure concentrations, Fig. 4a). With a significant increase of 30% (in cells exposed to 250 µg/mL of CBNP) as compared to control (Fig. 4a). However, after 24 h of exposure, a slight depolarization of the MMP was observed at lower exposure concentration (8 to 63 µg/mL), whereas at higher exposure concentration (125 and 250 µg/mL) of CBNP the mitochondria returned to the hyperpolarized state (Fig. 4b).

## Effect of CBNP exposure on mitochondrial ROS levels

It was observed that CBNP exposure in A549 led to mitochondria dysfunction. To analyze its influence on mitochondrial generated ROS, the fluorogenic dye MitoSox Red was used. As evident from the Fig. 4c, d, a concentration and time-dependent increase in mitochondrial ROS was observed. After 24 h exposure (Fig. 4d), an increase of up to 20% was already observed at lower exposure concentrations such as 4 µg/mL, while at higher concentration (250 µg/mL), a significant increase of 40% as compared to control was observed.

## Transcriptional profiling of oxidative stress-related genes in CBNP exposed cells in the presence and absence of W-7

From the experiments, it was observed a possible role of calcium-regulated protein CaM in CBNP-mediated oxidative stress. To translate this effect on the gene level, a PCR array analysis was performed in CBNP treated A549 cells. For this experiment, the samples were separated into two groups other than control. Group 1: A549 cell exposed to CBNP (125 µg/mL) and group 2: A549 cells pretreated with CaM inhibitor W-7.

In-group 1, 19% of the arrayed genes (*n* = 14) showed altered expression in the cells exposed to CBNP when compared to the control group (with a fold change regulation of ≥ 1.5-fold and a statistical cutoff at *P* < 0.05, Supplementary Table 2). The entire identified genes were found to be upregulated. Whereas, in group 2, 18 genes were found to be altered (with a fold change regulation of ≥ 1.5-fold and a statistical cutoff at *P* < 0.05, Supplementary Table 3). Among group 1 and 2 as compared to control, 14 genes involved in the antioxidant system and ROS metabolism were found to be regulated. The genes included members of glutathione peroxidase (GPX2, GPX4, CAT, PRDX5, SOD2, VIMP, PXDN, and MGST3) and ones involved in ROS metabolism (MPV17, UCP2, GTF2l, TXND2, CCL5).), whereas three genes (MPO, MT3, and NOS2) were specific for group 2. These genes have been identified for their role in inflammation, their upregulation in absence of calcium protein calmodulin indicates toward the role of calcium in cell protection upon CBNP-induced ROS toxicity.

**Fig. 5:**
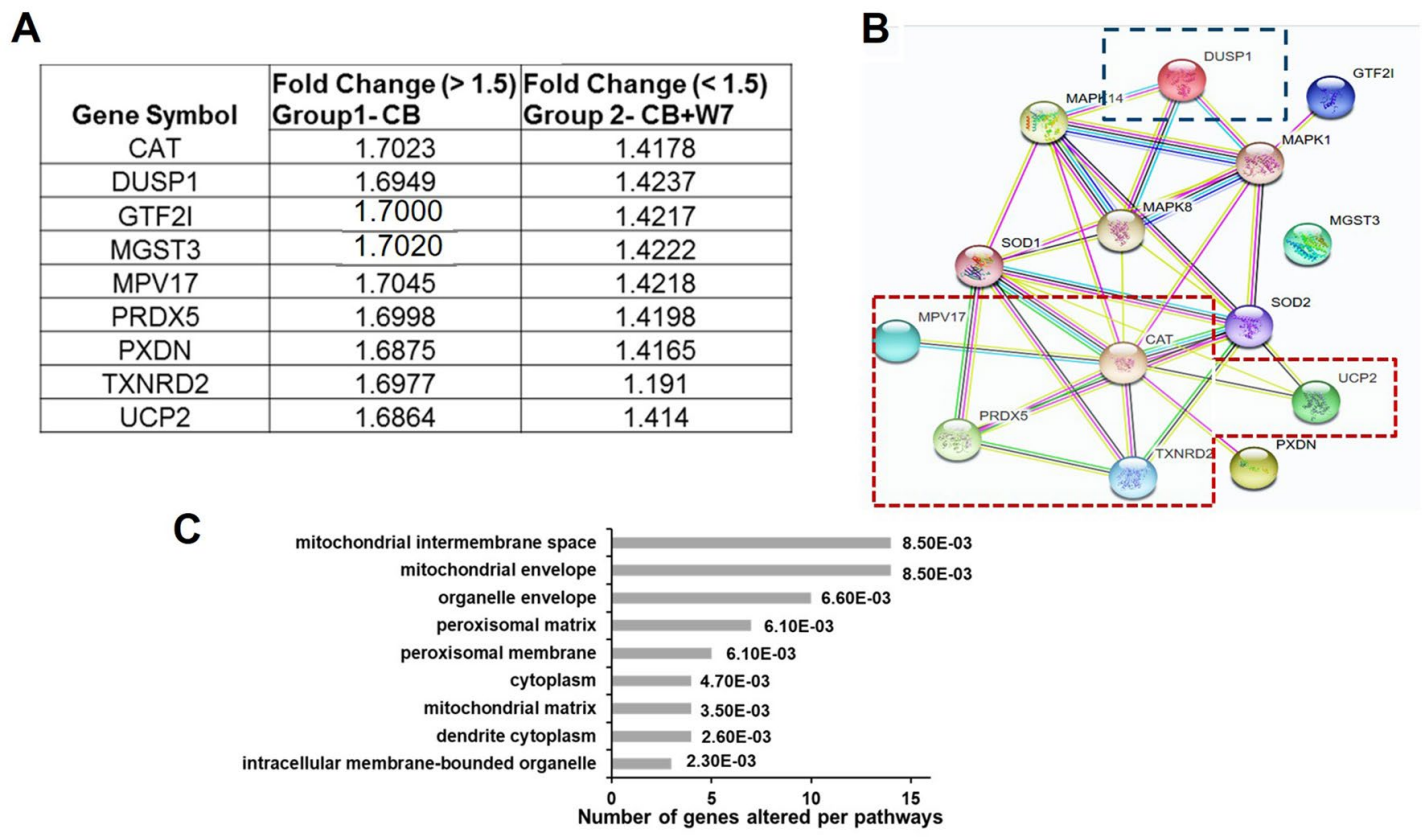
Gene expression profile of oxidative stress and antioxidant defense in lung cell line exposed to CBNP and CBNP + W7. **a** List of genes common in 2 groups (CBNP and CBNP + W7) with a fold changes > 1.5 in CB exposed cells and a fold change of < 1.5 in CB + W7. **b** Potential protein–protein interactions of all differentially expressed genes (*P* < 0.05) associated with CBNP exposure *vs* CBNP + W7 as suggested by the STRING 9 database and web resources. Gene names were loaded into the STRING tool (http://string-db.org/), and analyzed using the standard settings (medium confidence, network depth 1, no additional white nodes). The color of the connecting lines between two protein species encodes the source of the information: experimental data (rose), databases (light blue), co-expression data (black), co-occurrence data (dark blue), and text mining. (green) The nodes of interest are marked in colored boxes. **c** Cellular component enrichment analysis of screened proteins. The bar graph represents the number of genes enriched per cellular components of the cell. (*P < 0.05, FDR (false discovery rate) corrected)

To analyze the effect of CaM on CBNP gene regulation, a comparison of group 1 and group 2 was carried out. For comparison, only those genes were considered which showed a positive regulation (> 1.5) and a statistical cutoff at *P* < 0.05 in group 1 (w/o CaM), and no regulation in group 2 (< 1.5-fold, w/o CaM). Based upon the above criteria, a total of nine genes were further selected (Fig. 5a). The selected genes included catalase (CAT), dual specificity phosphatase 1 (DUSP1), general transcription factor IIi (GTF2l), microsomal glutathione S-transferase 3 (MGST3), mitochondrial inner membrane protein (MPV17), peroxiredoxin 5 (PRDX5), peroxidase homolog (PXDN), thioredoxin reductase 2 (TXDRD2) and uncoupling protein 2 (mitochondrial, proton carrier, UCP2). Network-based analysis to identify canonical pathways was carried out using the search tool STRING (Fig. 5b). Interestingly, the software associated the differentially expressed genes with a network containing the response to oxidative stress with most of the regulated genes concentrated to mitochondrial location (Fig. 5c). Another interesting pathway, which STRING analysis pointed during analysis, was the pathway related to mitogen-activated protein kinase (MAPK) signaling cascade. MAPKs are components of the signaling cascades known to initiate responses involved in cell growth, proliferation, and environmental stress (Cargnello and Roux 2011; Wada and Penninger 2004). The pathway was highlighted due to the alteration of gene DUSP1, which specifically dephosphorylates and inactivates MAPK. The downregulation of the above nine oxidative stress-related genes in absence of calcium suggests the specific contribution of Ca^2+^ (via generation of mitochondrial ROS) in CBNP-mediated oxidative stress.

## Discussion

The results of the present study provide an evidence that the disturbance in calcium homeostasis upon CBNP exposure in lung epithelial cells plays a pivotal role in generation of reactive oxygen species, and hence over all toxicity caused by CBNP exposure. The present hypothesis is supported by the findings that Ca^2+^ sequestration with calcium-regulated protein CaM and chelator BAPTA and verapamil, as all prevented the CBNP-induced ROS formation. Gene array analysis further supported these findings and revealed the mitochondrial dysfunction that the cells experience upon CBNP exposure.

## Disturbance in calcium homeostasis and the generation of oxidative stress

The generation of free radicals by CBNP has been well documented (Abdal Dayem et al. 2017; Madl et al. 2014; Manke et al. 2013). The unique surface chemistry, large surface area, and redox-active or catalytic contamination of nanoparticles are known to enable ROS generation (Fu et al. 2014; Gonzalez et al. 2008). CBNP exposure in A549 cells also showed a strong concentration-dependent increase in ROS levels, at nominal concentrations, at which 20 times high ROS levels were recorded as compared to control cells (Fig. 1a, b). Unfortunately, it was not possible to identify specific ROS species generated upon CBNP exposure in A459 cells; however, both antioxidants NAC and Trolox considerably inhibited ROS level, thus suggesting that their increase is an effect of different ROS species generated and is not dependent on an individual chemical species only. Other than ROS generation, CBNP exposure in A549 cells also exhibited a transient increase in calcium levels, as also observed in other studies (Holme et al. 2019). A slight disturbance in calcium homeostasis was observed already after 3 h of exposure, which increased consistently over 6 h, and remained consistent even after 24 h exposure. Incubation with calcium chelators BAPTA and verapamil decreased the calcium levels of the cells, indicating the involvements of both extra and intracellular calcium sources.

Additionally, to examine whether CBNP-induced disturbance in ROS and calcium were interrelated, ROS measurements were carried out in presence of calcium chelators BAPTA and verapamil. While a significant increase in ROS levels was observed in CBNP exposed cells, these were reduced by 32% in BAPTA and 30% verapamil pretreated cells, suggesting the involvement of calcium signaling in increased ROS levels as observed upon CBNP exposure. However, the pretreatment was not able to eliminate the ROS level to basal levels as compared to control cells, suggesting a contribution of another calcium-independent pathway in overall ROS levels upon CBNP exposure. Furthermore, pretreatment of cells with the antioxidants NAC and Trolox suppressed the ROS generation but were unable to modulate calcium signaling increased by CBNP exposure. The results of the study pointed toward the role of calcium signaling in ROS generation upon CBNP exposure.

## The interplay of calcium and ROS signaling system

Few studies have recognized a mutual and complex interplay between calcium and ROS signaling systems essential for the proper functioning of cellular signaling networks (Görlach et al. 2015; Hempel and Trebak 2017; Yan et al. 2006). It is now clear that sub-toxic levels of ROS act as signaling molecules, critical for various cellular processes including calcium signaling (Gordeeva et al. 2003; Görlach et al. 2015). By modulation of various extra and intracellular calcium channels and receptors, ROS can effectively modulate calcium signaling and hence its homeostasis (Feno et al. 2019). Calcium, on the other hand, is known to regulate several cellular functions, including the ones involved in the generation of ROS, particularly by mitochondria in the form of mitochondrial ROS (Adam-Vizi and Starkov 2010).

## CBNP-associated mitochondrial dysfunctions

Since mitochondria are an integral part of Ca^2+^-mediated signal transduction cascades, mitochondria can take up, buffer, and release Ca^2+^ ions to shape cytosolic Ca^2+^ transients, as well as stimulate ATP and mitochondrial ROS production (DeLuca and Engstrom 1961). Moreover, three-dimensional conformational changes in the respiratory chain complexes due to calcium ions have been reported to increase the generation of mitochondrial ROS (Brookes et al. 2004). Our initial studies revealed a substantial increase in ROS upon CBNP triggered by calcium. Also, a substantial amount of increase in mitochondrial calcium was observed (Fig. 1e, f). To analyze if CBNP exposure also led to mitochondrial dysfunction in these cell lines, MMP and mitochondrial ROS measurements were carried out. The investigation revealed hyperpolarization of mitochondria as soon as after 6 h of exposure in cells exposed to high CBNP (63 to 250 µg/mL) (Fig. 4a), which stayed even after 24 h (Fig. 4b). Interestingly, there are studies, suggesting that hyperpolarization of mitochondria is significantly influenced by the elevated cellular ROS levels. It is therefore likely that the CBNP-mediated increased ROS levels (especially at high concentrations, Fig. 1a) in A549 cells are responsible for the observed hyperpolarization of mitochondria. The elevated hyperpolarization is further known to cause ROS overproduction, which further leads to mitochondrial dysfunction and more ROS production, thus causing cell damage. The results are supported by a significant amount of mitochondrial ROS measured at all the exposure (nominal) concentrations (Fig. 4c, d).

To investigate further an underlying mechanism for the interaction of calcium and ROS in the mitochondria, we have focused on the role of calmodulin that is known to contribute to cellular dysfunction by promoting defective intracellular Ca^2+^ handling, including mitochondrial Ca^2+^ overload.

## The possible role of calmodulin in CBNP-induced oxidative stress and mitochondrial dysfunction

The increase in intracellular calcium concentration has been demonstrated to modulate cellular function by the activity of several calcium-binding proteins (Bagur and Hajnóczky 2017). Calmodulin is also one of such known proteins, forming complexes with calcium and thus participating in the regulation of several signal transduction pathways (Swulius and Waxham 2008). The first evidence for the role of calmodulin in oxidative stress in our study was the 50% reduction in ROS in cells pretreated with calmodulin.

To gain a deeper insight into the possible mechanism, a wide screening for 84 different genes involved in oxidative stress was performed in presence and absence of CaM inhibitor W-7. A set of nine genes were identified, which were upregulated after exposure to CBNP. However, upon pretreatment with the CaM inhibitor the same set of genes were found to be downregulated, indicating toward its role in the regulation of oxidative stress. Bioinformatic analysis of these nine genes via STRING analysis underlined among other the pathways involved in the response to oxidative stress. The mitochondria were highlighted as a main cellular component for most of the gene regulated (five genes i.e. CAT, MPV17, PRDX5, TXNRD2, and UCP2, Fig. 5c), thus supporting the gathered cytotoxicity data, exhibiting an increase in mitochondrial ROS, and hyperpolarization of mitochondria upon CBN nanoparticle exposure (Fig. 4). The ubiquitous protein CaM is found mainly in the cytoplasm, nucleus and plasma membrane, but few studies have also reported its presence in mitochondria. The role of calcium-activated protein CaM in mitochondrial dysfunction, which further leads to exacerbation of mitochondrial ROS, thus eventually leading to cell death, has been reported in few studies (Hajimohammadreza et al. 1995; Lee et al. 2005; Liu and Templeton 2007; Odagiri et al. 2009; Takano et al. 2003; Toledo et al. 2014). Regulation of the five mitochondrial proteins in the present study points toward the mitochondrial dysfunction that the cells undergo when exposed to the nanoparticles.

Another interesting observation was the regulation of the DUSP1 gene. Dual specificity phosphatases 1 (DUSP1) belongs to a family of stress-induced enzymes that plays an important role in feedback inhibition of mitogen-activated protein kinases (Caunt and Keyse 2013), one of the pathways highlighted during STRING analysis (Fig. 5).

MAPKs are components of the signaling cascades comprising extracellular signal-regulated kinase (ERK), c-Jun-NH2 kinase (JNK) and p38 by which these control a range of fundamental processes including inflammation and apoptosis (Cargnello and Roux 2011). MAPK signaling is also involved in the biological response to organic compounds, particularly carbon black (Donaldson et al. 2003; Dong and Ma 2015; Yuan et al. 2020).

DUSP1 seems to be part of a negative feedback loop controlling the immune response. It was reported that in A549 cells, IL-1β rapidly induced DUSP1 expression with subsequent inhibition of MAPKs and inflammatory gene expression (Shah et al. 2014). IL-1β is a key driver of acute inflammation after cellular damaging, by initiating downstream signaling together with TNF-α, it initiates production of chemokines by epithelial cells (Di Paolo and Shayakhmetov 2016). And it is quite conceivable that feedback mechanisms are activated at the same time to prevent an excessive immune response. Along with it, DUSP1 also appears to play a role in apoptosis due to oxidative stress (Jin et al. 2018). Further investigations are certainly necessary to elucidate the mode of action of calcium in nanoparticle-mediated ROS toxicity, but nevertheless these results underline the multifaceted role of calcium and calmodulin in the cellular response to CBNP.

## Conclusion

The modulation of intracellular calcium and oxidative stress upon exposure to CBNP has been observed in several studies. Increasing evidence suggests a mutual interplay between calcium and ROS signaling systems upon exposure to CBNP; however, the relevant pathways that contribute to such interplay remain poorly understood. With our study, it was presented that in CBNP exposed alveolar cells, calcium is the main driver of the biological response, as the changes in Ca^2+^ levels significantly reduced ROS formation. On the other hand, the reduction of ROS had no effect on calcium overload. The hypothesis was supported by studies on the role of calmodulin in this context.

For a better understanding as to how calcium and calcium-binding protein CaM regulates CBNP-mediated oxidative stress, an in-depth gene array analysis of genes involved in oxidative stress was performed in presence or absence of CaM. Interestingly, nine oxidative stress-related genes were found downregulated in absence of calcium-regulated gene CaM, therefore indicating the role of calcium-regulated CaM in CBNP nanoparticles mediated oxidative stress. Further, pathway analysis revealed five out of the nine identified genes were mitochondrial oxidative stress genes, hence pointing toward the role of mitochondria ROS upon CBNP exposure. The finding was further supported by the detection of a significant amount of mitochondrial ROS in CBNP exposed cells. The study thus points toward how CBNP-mediated lung toxicity is a result of both disturbances in calcium and ROS homeostasis, and thus be given full consideration while designing new strategies for its prevention.

## Supplementary Information

Below is the link to the electronic supplementary material.

Supplementary file 1 (DOCX 367 KB)
