# Supplementary material for: A new perspective on calmodulin-regulated calcium and ROS homeostasis upon carbon black nanoparticle exposure
Source: Arch Toxicol. 2021 Mar 27;95(6):2007–18. doi: 10.1007/s00204-021-03032-0 (PMC8166691; doi:10.1007/s00204-021-03032-0)
Supplement: Supplementary file 1 — (DOCX 367 KB) [file 204_2021_3032_MOESM1_ESM.docx]

**Supplementary Data**

**New perspective on calmodulin regulated calcium and ROS homeostasis upon carbon black nanoparticle exposure**

***Nisha Verma^1^*, Mario Pink^1^, and Simone Schmitz-Spanke^1^***

^1^Institute and Outpatient Clinic of Occupational, Social and Environmental Medicine, University of Erlangen-Nuremberg, Germany

*****Correspondence:**

Nisha Verma, Institute and Outpatient Clinic of Occupational, Social and Environmental Medicine, University of Erlangen-Nuremberg, Henkestrasse 9-11, D-91054 Erlangen, Germany

**E-mail:** nishaverma24@gmail.com

**Fax:** +49-9131-8522317

**Supplementary Figure 1:** **Alterations of cellular ROS and calcium homeostasis after exposure to CBN for 3 h** Cells (A549) were cultured on a clear bottom 96 well plates and exposed to 2-250 µg/ml of CBN for 3 h. For the analysis, the cells were loaded with fluorescent dyes H_2_DCFDA (10 μM for ROS) [A] Fluo-4/AM (1.4 µM and Rhod-2 AM (3.6 µM). for calcium measurements) [B-C], respectively for 30 min. The fluorescence was recorded by using a plate reader (Fluo 4/AM, λ_excitation_ = 488; Rhod-2/AM, λ_excitation_ = 543; λ_emission_ = 560 nm; λ_emission_ = 505 nm, H_2_DCFDA λ_excitation_ = 485; λ_emission_ = 535 nm). As evident from the graphs after 3 h exposure, no significant alterations were observed. The data were presented as mean ± standard deviation of four independent experiments with A549. The level of significance relative to the control was determined by using the t-test (*p <0.05).


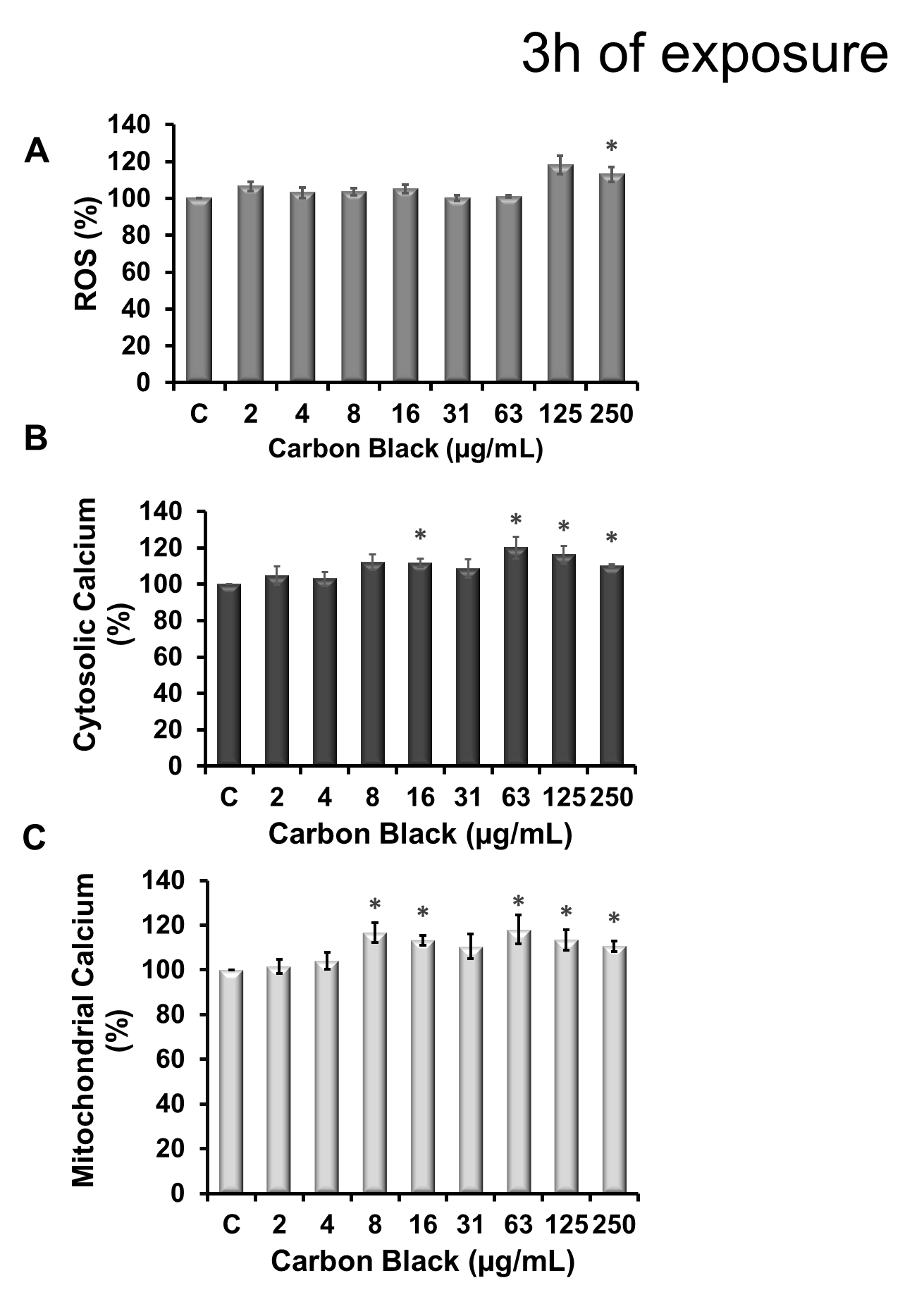


**Supplementary Fig. 2: Interaction between ROS and Ca^2+^ upon CB exposure** CB exposed cells showed increased levels of both ROS and intracellular Ca ^2+^ ions. To analyze a possible mutual interplay between two signaling system in our cell models we used antioxidants N-acetylcysteine (NAC) and trolox and Ca^2+^ pump inhibitors (verapamil) /chelators (BAPTA) as shown below.

**
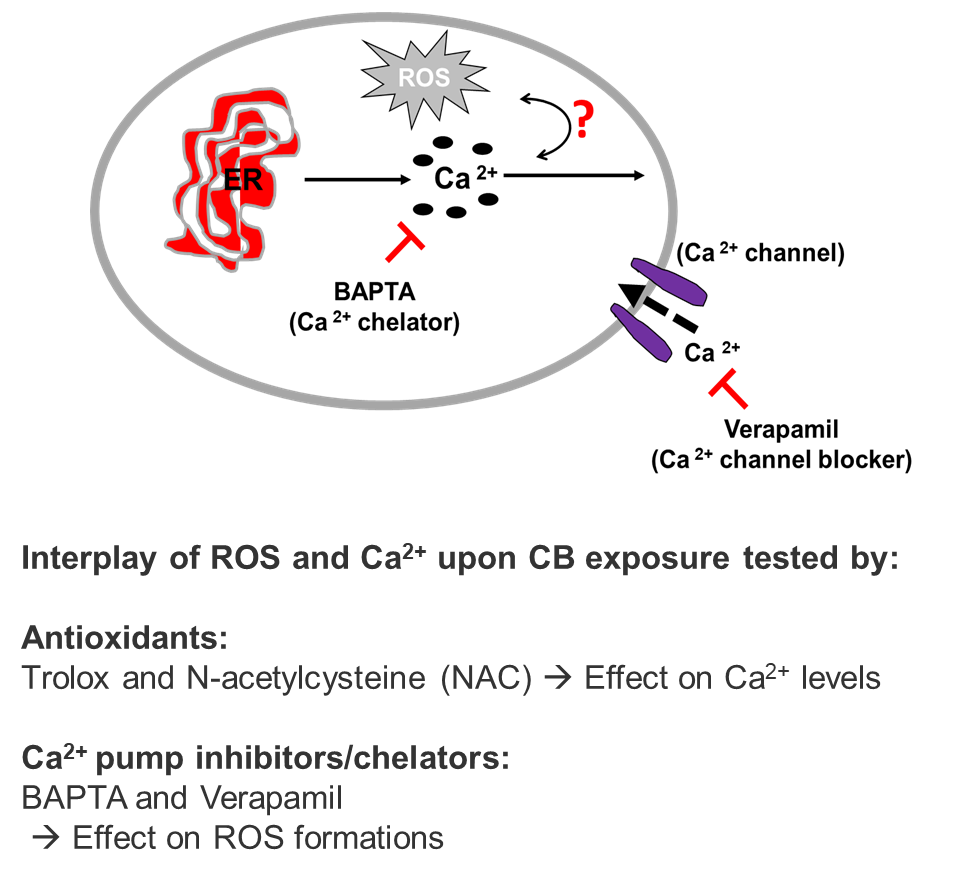
**

**Supplementary Figure 3:** **Changes in mitochondrial ROS and mitochondrial membrane potential (MMP) after exposure to CBN for 3 h.** Cells (A549) were cultured on a clear bottom 96 well plates and exposed to 2-250 µg/ml of CBN for 3 h. For MMP measurements, the cells were loaded with JC-1 dye (5 μM) [A]. Measurements were obtained immediately as a ratio of red aggregate of JC -1 dye with absorption/emission at 585/590 nm /green aggregate of the dye with absorption/emission of 510/527 nm in the mitochondria by using the Tecan microplate reader (Tecan, Mainz, Germany). For the mitochondrial ROS production MitoSOX Red (5 µM) (mitochondria-targeted superoxide indicator) dye was used. [B]. The fluorescence was recorded by using a plate reader (Mitosox, λ_excitation_ = 510; λ_emission_ = 580 nm). The data were presented as mean ± standard deviation of four independent experiments with A549  cells. The level of significance relative to the control was determined by using the t-test (*p <0.05).


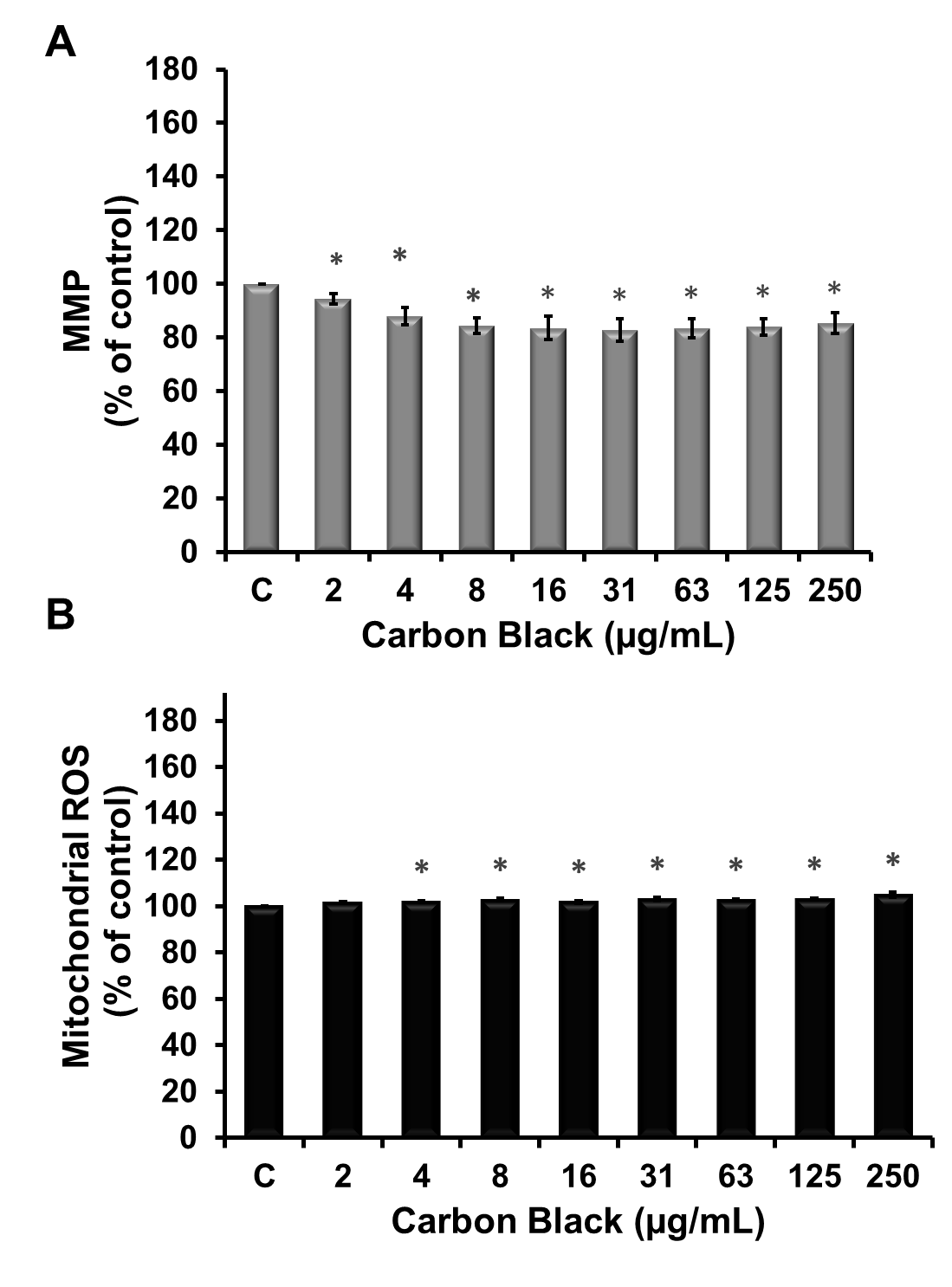


**Supplementary Fig.4**

Pathway Enrichment Analysis of the identified proteins using the STITCH software. The gene name was uploaded into the STITCH search engine that assigned it to different protein pathways. The bar graph represents the number of genes enriched per pathway

**.**

**
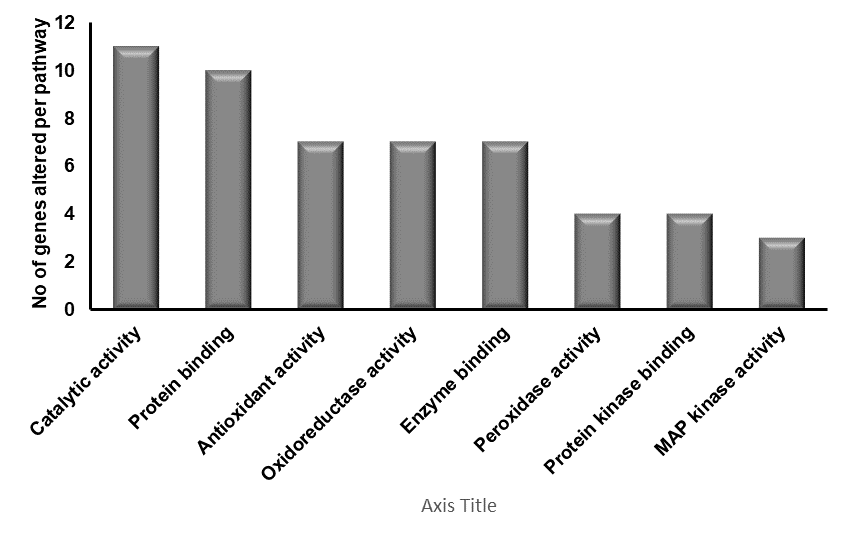
**

**Supplementary Table 1:** A complete list of 84 oxidative stress-associated genes used to analyze the transcriptional profiling of oxidative stress related genes in CB exposed cells. The Human Oxidative Stress Plus RT^2^ Profiler PCR array from Qiagen was used for the present study.

| **Position** | **Ref Seq Number** | **Gene Symbol** | **Description** |
| --- | --- | --- | --- |
| A01 | NM_000477 | ALB | Albumin |
| A02 | NM_000697 | ALOX12 | Arachidonate 12-lipoxygenase |
| A03 | NM_001159 | AOX1 | Aldehyde oxidase 1 |
| A04 | NM_000041 | APOE | Apolipoprotein E |
| A05 | NM_004045 | ATOX1 | antioxidant protein 1 homolog (yeast) |
| A06 | NM_004052 | BNIP3 | BCL2/adenovirus E1B 19kDa interacting protein 3 |
| A07 | NM_001752 | CAT | Catalase |
| A08 | NM_002985 | CCL5 | Chemokine (C-C motif) ligand 5 |
| A09 | NM_005125 | CCS | Copper chaperone for superoxide dismutase |
| A10 | NM_000397 | CYBB | Cytochrome b-245, beta polypeptide |
| A11 | NM_134268 | CYGB | Cytoglobin |
| A12 | NM_014762 | DHCR24 | 24-dehydrocholesterol reductase |
| B01 | NM_175940 | DUOX1 | Dual oxidase 1 |
| B02 | NM_014080 | DUOX2 | Dual oxidase 2 |
| B03 | NM_004417 | DUSP1 | Dual specificity phosphatase 1 |
| B04 | NM_001979 | EPHX2 | Epoxide hydrolase 2, cytoplasmic |
| B05 | NM_000502 | EPX | EPX Eosinophil peroxidase |
| B06 | B06 NM_021953 | FOXM1 | FOXM1 Forkhead box M1 |
| B07 | B07 NM_002032 | FTH1 | Ferritin, heavy polypeptide 1 |
| B08 | B08 NM_001498 | GCLC | Glutamate-cysteine ligase, catalytic subunit |
| B09 | B09 NM_002061 | GCLM | Glutamate-cysteine ligase, modifier subunit |
| B10 | B10 NM_000581 | GPX1 | Glutathione peroxidase 1 |
| B11 | NM_002083 | GPX2 | Glutathione peroxidase 2 (gastrointestinal) |
| B12 | NM_002084 | GPX3 | Glutathione peroxidase 3 (plasma) |
| C01 | NM_002085 | GPX4 | Glutathione peroxidase 4 (phospholipid hydroperoxidase) |
| C02 | NM_001509 | GPX5 | Glutathione peroxidase 5 (epididymal androgen-related protein) |
| C03 | NM_182701 | GPX6 | Glutathione peroxidase 6 (olfactory) |
| C04 | NM_015696 | GPX7 | Glutathione peroxidase 7 |
| C05 | NM_000637 | GSR | Glutathione reductase |
| C06 | NM_000178 | GSS | Glutathione synthetase |
| C07 | NM_000852 | GSTP1 | Glutathione S-transferase pi 1 |
| C08 | NM_001513 GSTZ1 | GSTZ1 | Glutathione transferase zeta 1 |
| C09 | NM_001518 GTF2I | GTF2I | General transcription factor IIi |
| C10 | NM_002133 | HMOX1 | Heme oxygenase (decycling) 1 |
| C11 | NM_005345 | HSPA1A | Heat shock 70kDa protein 1A |
| C12 | NM_006121 | KRT1 | Keratin 1 |
| D01 | NM_006151 | LPO | Lactoperoxidase |
| D02 | NM_005368 | MB | Myoglobin |
| D03 | NM_000242 | MBL2 | Mannose-binding lectin (protein C) 2, soluble |
| D04 | NM_004528 | MGST3 | Microsomal glutathione S-transferase 3 |
| D11 | NM_000625 | NOS2 | Nitric oxide synthase 2, inducible |
| D12 | NM_016931 | NOX4 | NADPH oxidase 4 |
| E01 | NM_024505 | NOX5 | NADPH oxidase, EF-hand calcium binding domain 5 |
| E02 | NM_000903 | NQO1 | NAD(P)H dehydrogenase, quinone 1 |
| E03 | NM_002452 | NUDT1 | Nudix (nucleoside diphosphate linked moiety X)-type motif 1 |
| E04 | NM_181354 | OXR1 | Oxidation resistance 1 |
| E05 | NM_005109 | OXSR1 | Oxidative-stress responsive 1 |
| E06 | NM_020992 | PDLIM1 | PDZ and LIM domain 1 |
| E07 | NM_007254 | PNKP | Polynucleotide kinase 3'-phosphatase |
| E08 | NM_002574 | PRDX1 | Peroxiredoxin 1 |
| E09 | NM_005809 | PRDX2 | Peroxiredoxin 2 |
| E10 | NM_006793 | PRDX3 | Peroxiredoxin 3 |
| E11 | NM_006406 | PRDX4 | Peroxiredoxin 4 |
| E12 | NM_181652 | PRDX5 | Peroxiredoxin 5 |
| F01 | NM_004905 | PRDX6 | Peroxiredoxin 6 |
| F02 | NM_020820 | PREX1 | Phosphatidylinositol-3,4,5-trisphosphate-dependent Rac exchange factor 1 |
| F03 | NM_183079 | PRNP | Prion protein |
| F04 | NM_000962 | PTGS1 | Prostaglandin-endoperoxide synthase 1 (prostaglandin G/H synthase and cyclooxygenase) |
| F05 | NM_000963 | PTGS2 | Prostaglandin-endoperoxide synthase 2 (prostaglandin G/H synthase and cyclooxygenase) |
| F06 | NM_012293 | PXDN | Peroxidasin homolog (Drosophila) |
| F07 | NM_014245 | RNF7 | Ring finger protein 7 |
| F08 | NM_182826 | SCARA3 | Scavenger receptor class A, member 3 |
| F09 | NM_203472 | VIMP | Selenoprotein S |
| F10 | NM_005410 | SEPP1 | Selenoprotein P, plasma, 1 |
| F11 | NM_003019 | SFTPD | Surfactant protein D |
| F12 | NM_012237 | SIRT2 | Sirtuin 2 |
| G01 | NM_000454 | SOD1 | Superoxide dismutase 1, soluble |
| G02 | NM_000636 | SOD2 | Superoxide dismutase 2, mitochondrial |
| G03 | NM_003102 | SOD3 | Superoxide dismutase 3, extracellular |
| G04 | NM_003900 | SQSTM1 | Sequestosome 1 |
| G05 | NM_080725 | SRXN1 | Sulfiredoxin 1 |
| G06 | NM_006374 | STK25 | Serine/threonine kinase 25 |
| G07 | NM_000547 | TPO | Thyroid peroxidase |
| G08 | NM_003319 | TTN | Titin |
| G09 | NM_003329 | TXN | Thioredoxin |
| G10 | NM_003330 | TXNRD1 | Thioredoxin reductase 1 |
| G11 | NM_006440 | TXNRD2 | Thioredoxin reductase 2 |
| G12 | NM_003355 | UCP2 | Uncoupling protein 2 (mitochondrial, proton carrier) |
| H01 | NM_001101 | ACTB | Actin, beta |
| H02 | NM_004048 | B2M | Beta-2-microglobulin |
| H03 | NM_002046 | GAPDH | Glyceraldehyde-3-phosphate dehydrogenase |
| H04 | NM_000194 | HPRT1 | Hypoxanthine phosphoribosyltransferase 1 |
| H05 | NM_001002 | RPLP0 | Ribosomal protein, large, P0 |
| H06 | SA_00105 | HGDC | Human Genomic DNA Contamination |
| H07 | SA_00104 | RTC | Reverse Transcription Control |
| H08 | SA_00104 | RTC | RTC Reverse Transcription Control |
| H09 | SA_00104 | RTC | Reverse Transcription Control |
| H10 | SA_00103 | PPC | Positive PCR Control |
| H11 | SA_00103 | PPC | Positive PCR Control |
| H12 | SA_00103 | PPC | Positive PCR Control |

**Supplementary Table2**: List of genes showing differentially altered expression in cell exposed to CB when compared with the control group (with a fold change regulation of ≥ 1.5-fold and a statistical cutoff at *P* ⩽ .05)

**Control vs Group 1**

| **Position** | **Symbol** | **Fold Change** | **P-value** |
| --- | --- | --- | --- |
| A07 | CAT | 1.70 | 0.021966 |
| A08 | CCL5 | 2.86 | 0.031665 |
| B11 | GPX2 | 1.69 | 0.021280 |
| C01 | GPX4 | 1.69 | 0.023706 |
| D04 | MGST3 | 1.70 | 0.024464 |
| D06 | MPV17 | 1.70 | 0.022502 |
| E12 | PRDX5 | 1.70 | 0.021400 |
| F05 | PTGS2 | 4.03 | 0.024853 |
| F09 | VIMP | 1.70 | 0.022478 |
| G02 | SOD2 | 1.68 | 0.023694 |
| G12 | UCP2 | 1.69 | 0.024535 |

**Supplementary Table 3**: List of genes showing altered expression in cell pre-exposed to CaM inhibitor W-7 for one hour followed by CB (125 µg/ml) exposure for another 24 h, when compared with the control group (with a fold change regulation of ≥ 1.5-fold and a statistical cutoff at *P* ⩽ .05)

**Control VS Group 2**

| **Position** | **Symbol** | **Fold Change** | **P value** |
| --- | --- | --- | --- |
| A08 | CCL5 | 4.03 | 0.000109 |
| B11 | GPX2 | 1.68 | 0.023139 |
| C01 | GPX4 | 1.69 | 0.025359 |
| D05 | MPO | 8.03 | 0.043211 |
| D08 | MT3 | 5.67 | 0.049764 |
| D11 | NOS2 | 3.37 | 0.039602 |
| F04 | PTGS1 | 2.38 | 0.031400 |
| F09 | VIMP | 1.69 | 0.023559 |
| G02 | SOD2 | 1.68 | 0.023932 |
